# Development of a *waxy* gene real-time PCR assay for the quantification of sorghum waxy grain in mixed cereal products

**DOI:** 10.1186/s12896-015-0134-z

**Published:** 2015-03-19

**Authors:** Jaemin Cho, Taewook Jung, Jungin Kim, Seokbo Song, Jeeyeon Ko, Koansik Woo, Jaesaeng Lee, Myeongeun Choe, Inseok Oh

**Affiliations:** Coarse Cereal Crop Research Division, National Institute of Crop Science, Miryang, Gyeongnam 627-803 Republic of Korea

**Keywords:** *waxy*-grain sorghum, *wx*^*a*^ allele, Allele-specific primer, qPCR

## Abstract

**Background:**

Waxy-grain sorghum is used in most of the commercial cereal products in Korea. Worldwide, three *waxy* mutant alleles have been identified in the sorghum germplasm, and DNA markers for these alleles have been developed to identify the *waxy* genotype. However, that detection method cannot be used to determine the proportion of waxy content in samples containing both waxy and non-waxy sorghum. This study developed an assay that can be used to detect and quantify the *waxy* content of mixed cereal samples.

**Results:**

All Korean waxy-grain sorghum used in this study contained the *wx*^*a*^ allele, and one *wx*^*a*^ allele-containing individual was also heterozygous for the *wx*^*c*^ allele. No individuals possessed the *wx*^*b*^ allele. The genotyping results were confirmed by iodine staining and amylose content analysis. Based on the sequence of the *wx*^*a*^ allele, three different types of primers (*wx*^*a*^ allele-specific, non*-waxy* allele-specific, and nonspecific) were designed for a quantitative real-time PCR (qPCR) assay; the primers were evaluated for qPCR using the following criteria: analytical specificity, sensitivity and repeatability. Use of this qPCR assay to analyze mixed cereal products demonstrated that it could accurately detect the *waxy* content of samples containing both waxy and non-waxy sorghum.

**Conclusions:**

We developed a qPCR assay to identify and quantify the *waxy* content of mixed waxy and non-waxy sorghum samples as well as mixtures of cereals including sorghum, rice and barley. The qPCR assay was highly specific; the allele-specific primers did not amplify PCR products from non-target templates. It was also highly sensitive, detecting a tiny amount (>0.5%) of *waxy* sorghum in the mixed samples; and it was simple and repeatable, implying the robust use of the assay.

**Electronic supplementary material:**

The online version of this article (doi:10.1186/s12896-015-0134-z) contains supplementary material, which is available to authorized users.

## Background

Waxy-grain sorghum constitutes most of the sorghum grown in Korea, reflecting its wide use in dishes such as steamed kernels with rice as well as in traditional foods (‘bukkumi’ – pan-fried cake, and ‘tteok’ – steamed or pounded cake) and beverages (‘munbaeju’ – distilled liquor). Non-waxy-grain sorghum is also used for human consumption (in noodles and confections), but it is mainly used for animal feed [1-3]. The waxy and hetero-waxy sorghum grain phenotypes are defined by endosperm starch containing 0-15% amylose and 85-100% amylopectin [4]. The scarcity or absence of amylose may result in higher starch and protein digestibility, which is associated with reduced kernel density and increased space between the starch granules [5]. In addition, the waxy grains were shown to have higher ethanol yield and greater conversion efficiency than the non-waxy grains, implying better quality for sorghum processing [6,7].

In sorghum (*Sorghum bicolor* (L.) Moench), waxy grains are caused by the loss of granule-bound starch synthase I (GBSS I) activity, leading to starch granules that contain primarily amylopectin and little or no amylose. The sorghum *GBSS I* gene is composed of 14 exons and small introns; exon 1 is untranslated, and exons 2–14 contain the coding sequence. The loss of GBSS I activity can be explained by mutations in its gene sequence; three *waxy* (*wx*) mutant alleles have been identified from waxy grain sorghum lines [8] and from a Taiwanese landrace [9]. The *wx*^*a*^ allele contains a large insertion in the third exon, the *wx*^*b*^ allele has a missense mutation that changes glutamine 268 to a histidine, and the *wx*^*c*^ allele consists of a +1G-to-C mutation in the 5′ splice site at the intron 10-exon 11 boundary [9,10]. To differentiate between the *waxy* and non-*waxy* alleles of the *GBSS I* locus, allele-specific DNA markers were developed. The *wx*^*a*^-specific marker was designed based on the sequence of the insertion and amplifies a 615-bp fragment from the *wx* allele, whereas the *wild-type* allele-specific primer amplifies a 523-bp fragment from the non-*waxy* allele. The *wx*^*b*^ PCR product contains a single *Nco*I restriction site that can be digested to generate two DNA fragments. The wild-type PCR product, which lacks the *Nco*I site, still contains a single fragment after digestion [5]. *wx*^*c*^-specific primers were designed to detect only the *wx*^*c*^ allele, and the primers for the wild type detected the *wild-type* allele [9].

PCR-based DNA markers have been used to screen the germplasm to find new *waxy* alleles in accessions with *waxy* phenotypes but lacking known mutant alleles. By screening waxy Chinese sorghum accessions with the *wx*^*a*^ and *wx*^*b*^ markers, Lu et al. [11] identified two novel types of *waxy* mutations and developed cleaved amplified polymorphic sequence markers based on these new alleles to classify their germplasm. In this study, to determine whether a collection of Korean sorghum varieties and landraces contained any unknown types of *waxy* mutations, the *waxy* alleles in the collection were investigated using three DNA markers (*wx*^*a*^, *wx*^*b*^ and *wx*^*c*^). We found that all of the waxy varieties and landraces in the collection contained the *wx*^*a*^ allele, implying that only this mutation was maintained during the domestic selection of Korean sorghum.

An inspection of the ingredients lists of sorghum-containing products in Korean grocery stores shows that these products contain only waxy sorghum, but there is no way to visually determine whether any non-waxy grain is also present. PCR-based DNA markers can be used to detect the presence of waxy grain or powder in commercial products. However, neither DNA markers nor any other currently available method can be used to determine the proportion of waxy grain in a mixed-grain product. Fluorescence-based quantitative real-time PCR (qPCR) has the capacity to detect and measure minute amounts of nucleic acids in a wide range of samples from numerous sources [12,13], and it can detect and differentiate between closely related species [14,15]. Here, we report a method that uses qPCR and the *waxy* gene (specifically the *wx*^*a*^ allele) to quantify the *waxy* content of various samples.

## Results

### *waxy* alleles in Korean sorghum varieties and landraces

**Figure 1:**
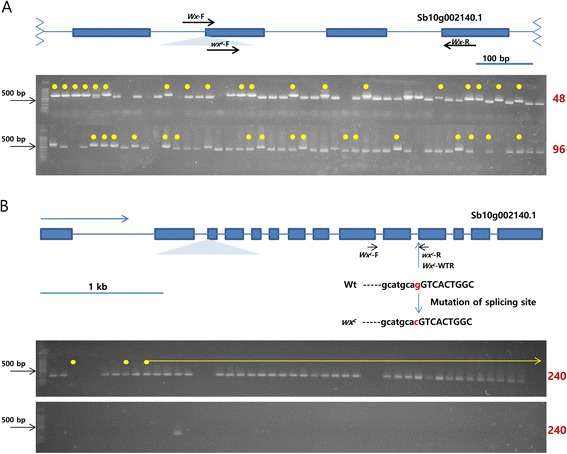
**Genotypic analysis of Korean sorghum varieties and landraces and foreign sorghum using** ***waxy*** **markers.** Yellow dots and arrows indicate Korean sorghum collections. Numbers in maroon indicate the identification number of the sample at the end of each electrophoregram. **(A)** The location of the *wx* ^*a*^ primer is shown on the *GBSS I* gene diagram. In the 3% agarose gel, the upper band (615 bp) indicates the *wx* ^*a*^ mutant allele, whereas the lower band (523 bp) indicates the *wild-type* allele. **(B)** The location of the *wx* ^*c*^ primer is shown on the *GBSS I* gene diagram. The *wild-type* (upper panel) and *wx* ^*c*^ mutant alleles (lower panel) are indicated by a single 305-bp band. One individual has both a *wild-type* allele and a mutant *wx* ^*c*^ allele.

A set of three PCR-based markers derived from three *waxy* alleles was synthesized to identify the *wx*^*a*^, *wx*^*b*^, *wx*^*c*^ and *wild-type GBSS I* alleles in various Korean sorghum varieties and landraces. The results of genotyping using the PCR-based markers for *wx*^*a*^, *wx*^*b*^ and *wx*^*c*^ should correlate with the waxy phenotype. The *waxy* genotype for each of the individual Korean sorghum varieties and landraces was determined via PCR for each of the three *waxy* allele markers. A total of 384 individuals, including 175 Korean sorghum accessions from the sorghum collection, were subjected to PCR amplification. Most of the accessions that were foreign or of unknown origin contained the *wild-type* allele (187 of 209 accessions [89.5%]; 4 accessions [1.9%] yielded no data) and the remaining 18 accessions (8.6%) contained the *wx*^*a*^ allele. Among the 175 tested Korean accessions, 150 (86%) contained the *wx*^*a*^ allele, and 25 (14%) had no *waxy* alleles. One foreign accession had the *wx*^*c*^ allele (*wx*^*c*^/*wx*^*c*^). One Korean individual was heterozygous for the *wx*^*c*^ allele (*Wx*/*wx*^*c*^), and it also possessed the *wx*^*a*^ allele (Figure 1 and Additional file 1). No *wx*^*b*^ individuals were found in any of the accessions. This result suggested that all waxy-grained Korean sorghum was the result of one *waxy* allele (*wx*^*a*^) in the genome.

### Iodine staining and amylose content of the endosperm

**Figure 2:**
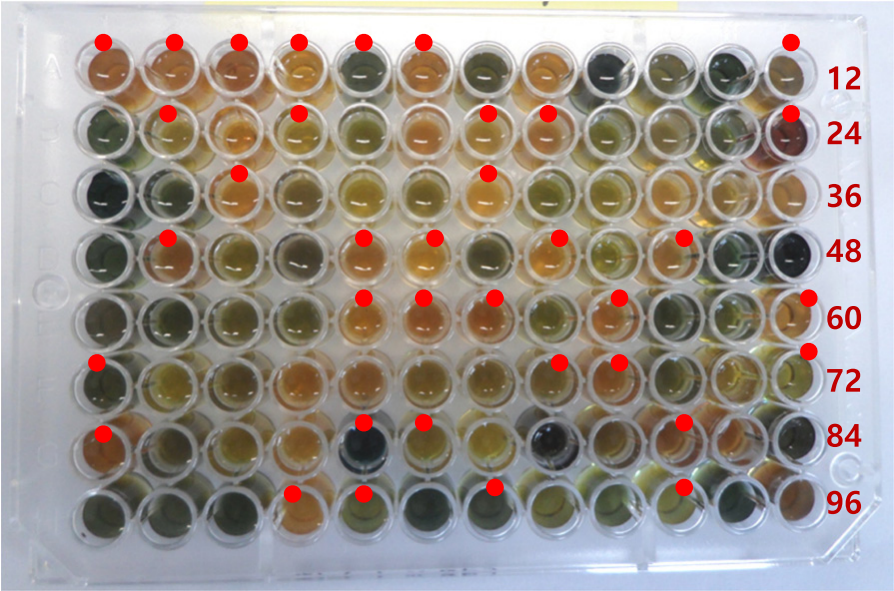
**Iodine test of Korean sorghum varieties and landraces and foreign sorghum.** Red dots indicate Korean sorghum collections. The colorimetric reactions were scored as follows: red for *waxy* and green for non-*waxy*. Each well represents one independent line. Numbers in maroon indicate the identification number of the sample at the end of each row.

To confirm the *waxy* allele data, the waxy and non-waxy grains of all individuals were stained with iodine (0.2% I_2_—2% KI), and the color of the endosperm was scored: waxy endosperm was reddish-brown, and non-waxy endosperm was dark blue. Of the 175 Korean sorghum accessions examined, iodine stained 25 of them pale or dark blue, showing that they were positive for the presence of amylose, whereas 150 accessions exhibited the waxy phenotype and stained pale or dark reddish-brown (Figure 2). All of the iodine test scores matched the *waxy* allele genotypes determined via DNA marker analysis, confirming that there were no new *waxy* alleles in the individuals that were wild type for the three *waxy* PCR-based markers.

**Figure 3:**
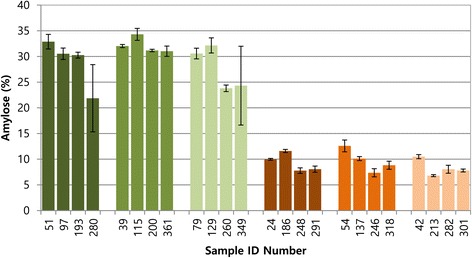
**The amylose content of waxy and non-waxy grain samples.** Based on the results of the iodine test, six sets of samples were chosen. The members of each group had similar results in the iodine test. The dark green, green and pale-green bars represent the non-waxy grains, and dark red, red and pale red bars represent the waxy grains. Each bar represents the mean amylose content as calculated from three replicate measurements. The error bars represent standard deviation.

To determine whether the degree of the iodine reaction was related to amylose content, the amylose content of twenty-four individuals with various reactions to the iodine solution (dark, medium, or pale blue or red) was measured. The individuals that stained blue had amylose content that ranged from 14.30 to 35.60%, whereas those of the individuals that stained red ranged from 6.59 to 13.84%. The accessions that stained dark blue in the iodine test contained >21.87% amylose, with an average of 28.89%; medium blue individuals contained >31.02% amylose and averaged 32.13%; and pale blue individuals contained >23.80% amylose and averaging 27.71%. By contrast, the accessions that stained dark red in the iodine test contained <11.60% amylose and averaged 9.35%; medium red individuals contained <12.59% amylose and averaged 9.72%; and pale red individuals contained <10.49% and averaged 8.28%. There were no significant differences in the amylose contents of individuals that stained the same color but to different degrees, but there was a significant difference between the blue and red groups (Figure 3), suggesting that the varied color intensity could be caused by other factors such as temperature or the presence of water-miscible organic solvents.

### Primer design, PCR specificity, and qPCR sensitivity

**Figure 4:**
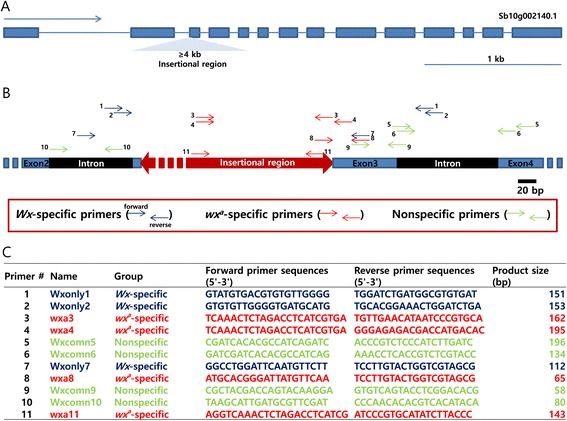
**Primer sequences and locations in the** ***GBSS I*** **gene containing an insertion within exon 3. (A)** Schematic of the waxy gene (Sb10g002140) showing the insertion. **(B)** The red portion of the schematic indicates the insertion. Each short arrow designates a primer location, and the different colors represent different specificities: blue, *Wx*-specific; red, *wx* ^*a*^-specific; and green, nonspecific. **(C)** The primer sequences are listed in the table.

In addition to the three allele-specific markers, eleven forward and eleven reverse primers were designed to identify the most efficient primer pairs for the qPCR-based assay. To selectively amplify products from waxy and non-waxy plants, allele-specific primers for the *waxy* and non-*waxy* alleles, as well as nonspecific primers, were designed based on a partial sequence of the *GBSS I* gene (starting from the end of exon 2 to the beginning of exon 4 and including part of the insertion) (Figure 4). To evaluate the PCR specificity, all possible forward and reverse primer combinations were tested on *waxy* and non-*waxy* DNA samples and on a no-template control (NTC). Among the sixteen *waxy*-specific combinations, the *wx*^*a*^ 11 forward and *wx*^*a*^ 8 reverse primer pair amplified a product with stable C_T_ values, and it did not produce spurious products from the non-*waxy* DNA samples or the NTC. By these criteria, the second and third most specific primer pairs were *wx*^*a*^ 11 forward with *wx*^*a*^ 11 reverse and *wx*^*a*^ 4 forward with *wx*^*a*^ 8 reverse. The other primer combinations either non-specifically amplified the samples or did not amplify a product at all. Using the same criteria for PCR specificity, two primer pairs, the *Wx*only7 forward primer with either the *Wx*only1 or *Wx*only2 reverse primers, were chosen from nine non-*waxy* allele-specific primer combinations. Out of sixteen non-specific primer combinations, the *Wx*comn9 forward primer with either the *Wx*comn5 or *Wx*comn6 reverse primers produced products from both *waxy* and non-*waxy* DNA samples but not from the NTC. Additional PCR reactions were performed and the most efficient primer pair for each sample type was determined: *wx*^*a*^ 11 with *wx*^*a*^ 8 for *waxy* samples, *Wx*only7 with *Wx*only2 for non-*waxy* samples and *Wx*comn9 with *Wx*comn6 for both types of samples.

**Figure 5:**
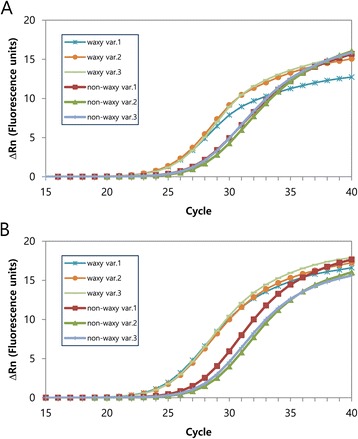
**qPCR amplification plots of different waxy and non-waxy varieties. (A)** The plots were generated via qPCR with the *wx* ^*a*^-specific primer pair (11 forward/8 reverse) on the waxy varieties and the *Wx*only primer pair (7 forward/2 reverse) on the non-waxy varieties. **(B)** The plots were generated based on the results of qPCR using the nonspecific primer pair (9 forward/6 reverse) on both types of sorghum.

The primer specificity was further evaluated by amplifying DNA samples from three waxy and three non-waxy sorghum varietals using the selected primer pairs described above. The amplification plots of the waxy individuals nearly completely overlapped, and the same was true for the non-waxy individuals (Figure 5). A single, group-specific peak was observed in the dissociation curves, ruling out the possibility of non-specific amplification. DNA fragments of the expected sizes were also confirmed through 3% agarose gel electrophoresis.

To determine the qPCR sensitivity and detection limits using the three primer pairs, DNA samples obtained from waxy and non-waxy individual plants and grains were two-fold serially diluted from 100.0 ng to 3.05 pg, and the samples were used to construct standard curves (Additional file 2). The linear correlation coefficients (*R*^*2*^) were calculated, and they ranged from 0.991 to 0.998. The curves for all three primer pairs were linear over a range of 100.0 ng to 12.21 pg, and the detection limit of all three primer pairs was approximately 12.0 pg. Below the detection limit, the plots were no longer linear due to overlapping positive signals from the DNA samples and the non-template controls (C_T_ values of approximately 35). This phenomenon was likely due to an accumulation of primer dimers to which the SYBR Green bound. The dissociation curves for the non-specific products were different from those of the correct amplicons (results not shown). The C_T_ values of the no-template controls overlapped with the C_T_ values obtained from DNA dilutions that were below the detection limit.

### The detection of waxy sorghum in mixed samples

To test the ability of the selected waxy primer pair to detect low proportions of waxy material in mixed samples containing both waxy and non-waxy sorghum powder, a set of reference mixtures of grain containing different proportions of waxy sorghum was prepared by manual grinding. DNA obtained from these reference samples was subjected to the qPCR assay, the waxy and non-waxy C_T_ values in the samples were interpolated on the standard curves defined in Additional file 2, and the DNA ratios thus obtained were compared with the actual ratios of waxy and non-waxy grain present in the mixtures. The ΔC_T_ was also calculated by subtracting the C_T*Wx*_ of the *Wx*-specific primer reaction from the C_T*wx*_^*a*^ of the *wx*^*a*^-specific primer reaction for each reference sample. The results showed a linear correlation between the actual and calculated proportions (Table 1 and Table 2). The ΔC_T_ calculation allowed an estimation of the ratio of *waxy* versus non-waxy primer reaction products by taking the difference between C_T*wx*_^*a*^ and C_T*Wx*_, as in [14]. Table 1 and Table 2 show the results obtained by calculating ΔC_T_, which was inversely correlated with the proportion of the *waxy* content of the mixtures. The *wx*^*a*^-specific primers generated an unstable signal when the *waxy* content was less than 0.5% or when the ΔC_T_ value was negative (in the case of no signal) or greater than 10 cycles (in the case of primer dimers, whose C_T_ value was approximately 35). Thus, the detection limit of the *waxy* allele using this qPCR method is 0.5%, which is the level required to produce a stable, measurable signal from the mixed samples.

**Table 1:** **The observed C**_**T**_ **differences and the calculated DNA concentrations (N) based on qPCR assays (DNA mix)**

**% waxy DNA**	**DNA mix**						
	**C**_**T**_ ***wx*** ^***a***^	**C**_**T**_ ***Wx***	**ΔC**_**T**_ ***wx*** ^***a***^ ***-Wx***	**ΔC**_**T**_ **STD**	**N** ***wx*** ^***a***^	**N** ***Wx***	**Calculated % waxy DNA (R** ^**2**^ **= 0.9991)**
100	24.3	36.0	−11.7	-	4.5	0.0	99.8
50	25.2	27.7	−2.5	0.0	2.4	2.4	49.7
20	26.7	26.9	−0.2	0.4	0.9	4.1	18.2
10	27.4	26.6	0.8	0.2	0.6	4.9	10.1
5	28.6	27.2	1.4	1.1	0.2	3.4	6.8
2	29.5	26.9	2.6	0.5	0.1	4.3	3.2
1	30.6	26.6	4.0	0.7	0.1	5.1	1.3
0.5	32.0	26.4	5.6	0.5	0.03	5.7	0.5
0	-	26.1	−26.1	0.2	0.0	7.1	0.0

The qPCR reactions were performed using 5 ng of DNA mix or DNA extracted from grain mix.

Mean and standard deviation (STD) values from three independent assays.

**Table 2:** **The observed C**_**T**_ **differences and the calculated DNA concentrations (N) based on qPCR assays (Grain mix)**

**% waxy DNA**	**Grain mix**						
	**C**_**T**_ ***wx*** ^***a***^	**C**_**T**_ ***Wx***	**ΔC**_**T**_ ***wx*** ^***a***^ ***-Wx***	**ΔC**_**T**_ **STD**	**N** ***wx*** ^***a***^	**N** ***Wx***	**Calculated % waxy DNA (R** ^**2**^ **= 0.9918)**
100	24.1	37.7	−13.6	-	4.9	0.0	99.9
50	24.1	26.1	−2.0	0.0	5.0	7.2	40.8
20	24.9	25.1	−0.2	0.1	3.0	13.1	18.4
10	26.2	25.4	0.8	0.3	1.3	11.4	10.1
5	28.1	25.6	2.5	0.6	0.3	10.0	3.4
2	28.7	25.6	3.1	0.2	0.2	9.7	2.3
1	30.3	26.3	4.0	0.5	0.1	6.1	1.4
0.5	29.1	25.2	3.9	0.1	0.2	13.1	1.4
0	-	25.8	−25.8	0.2	0.0	8.3	0.0

The qPCR reactions were performed using 5 ng of DNA extracted from each grain mixture.

The results are the means and standard deviations (STDs) of three independent assays.

### The detection of waxy content in commercial samples

**Figure 6:**
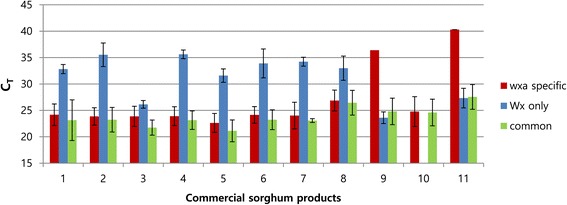
**qPCR assay on commercial sorghum products.** Numbers 1 through 9 indicate commercial sorghum products. Numbers 10 and 11 represent control *waxy* and non-*waxy* DNA, respectively. Each bar represents the mean C_T_ value calculated from three replicate measurements. The missing bar indicates that no signal was detected. The error bars indicate standard deviation.

The detection of *waxy* and non-*waxy* DNA in commercial cereal products can be further complicated by the presence of various cereals, and the PCR reactions could be hindered by the presence of inhibitors in the DNA extract. To test the behavior of the qPCR system and the selected primer pairs on commercial samples, PCR reactions were run on nine different commercial sorghum brands: seven sorghum grain products containing only waxy grains and two sorghum powder products with unknown sorghum content declared on the package label. DNA was extracted from all of the products, and the amplification of *waxy* DNA was tested using the three selected primer pairs. The reactions for products 1, 2, and 6 through 8 suggested no or <0.5% non-*waxy* grain in these products. Product 3 generated products for both waxy and non-waxy grains, and product 9 generated a PCR product only with the non-waxy primers (Figure 6). To determine whether the PCR reactions were inhibited in the samples obtained from the commercial grains and powders, the reactions were compared with control reactions using *waxy* (10 in Figure 6) and non-*waxy* (11 in Figure 6) DNA. No inhibition was observed in these tests, suggesting that the results from the commercial grain and powder products corresponded to the amplifiable DNA present in the extracts. It is worth noting that the qPCR assay and primer pairs suggested the presence of non-waxy grains in product 3. Because the 12.0 pg detection limit for *waxy* and non-*waxy* DNA was determined by standard curve analysis, it is reasonable to hypothesize that grain samples containing >0.5% *waxy* DNA would yield a positive signal from the *wx*^*a*^-specific primer pair in reactions containing 5 ng of total grain DNA regardless of the *waxy* DNA content. Based on the ΔC_T_ value (negative 2.27), the sorghum content of product 3 was approximately 50% non-*waxy*.

## Discussion

A rapid and sensitive qPCR-based method to detect *waxy* sorghum DNA was developed to provide stakeholders with a tool to evaluate the quality of sorghum products. To the best of our knowledge, tools to quantify waxy-grain sorghum material have not been developed to date. Therefore, we have developed a technique to detect and quantify the amount of *waxy* sorghum DNA in a mixed sample. Our method could be applied to calculate the ratio of waxy to non-waxy materials, which would be particularly informative for cereal processing. Thus, the widespread application of qPCR analysis to quantify *waxy* sorghum DNA is needed for sorghum processing and quality control.

The *waxy* sorghum genotypes can be classified based on three mutant alleles, which can be detected using specific DNA markers that are based on their sequences [5,9]. The Korean sorghum germplasm includes more than 1600 landraces that were collected from all of the counties in South Korea and are maintained at the RDA Genebank. In this study, we sampled approximately 10% of all catalogued Korean landraces and analyzed the frequency of the *waxy* alleles using three mutant allele-specific DNA markers. Each individual was a randomly selected representative of each city or county in the nation. Because Korean sorghum landraces have narrow gene pools, as shown by genetic diversity analysis with SSR markers (data not shown, manuscript in preparation), one random sample per city or county should be sufficient to investigate the *waxy* alleles. Six varieties of Korean sorghum breeding lines were also included in this study because they are the only widely distributed sorghum germplasm in the country. All tested Korean landraces and breeding materials that had *waxy* genotypes contained the *wx*^*a*^ allele, and one of these *wx*^*a*^ landraces was heterozygous for the *wx*^*c*^ allele. There was no indication of the existence of a new allele in the Korean sorghum germplasm and breeding materials because the genotypic and phenotypic data (determined by iodine staining and amylose content) matched perfectly. Thus, we concluded that the *wx*^*a*^ allele could be a target for the detection and quantification of the *waxy* content in mixed samples.

Optimization of the qPCR assay was critical to its successful use in the detection of *waxy* materials. Both the primer and the target sequences can affect the efficiency, specificity and accuracy of PCR assays [16]. We designed novel sets of primers based on three different allele-specific DNA markers from the region flanking the insertion in the *GBSS I* gene. To provide ideal qPCR assay conditions, the primers were designed to amplify small (<200 bp) PCR products, as per the guidelines of [17]. Out of all possible primer pair combinations, the selected primer pairs showed good specificity in each type of sorghum plant; the *waxy*-specific primers did not amplify products from non-*waxy* sorghum plants and vice versa. The nonspecific primer pair amplified products from both types of sorghum. Moreover, standard curves constructed using different waxy and non-waxy sorghum varieties showed similar amplification efficiencies, further supporting the specificity of the primers in calculating the proportion of *waxy* DNA. It should be mentioned that BLAST analysis of the primer sequences generated hits against only the sorghum *GBSS I* gene, and the analysis showed 100% identity between the sequences of the *GBSS I* gene and the primers.

The sensitivity (detection limit) of the qPCR assay can be used as another criterion to indicate its robustness. Standard curves generated using DNA from waxy sorghum, non-waxy sorghum, and mixtures of both demonstrated that our qPCR analysis could efficiently measure as little as 12.0 pg of DNA. The amplification cycle of non-waxy sorghum was one cycle greater than that of waxy sorghum, and the difference in C_T_ values between the waxy and non-waxy PCR products might be attributed to the characteristic curves of the samples’ amplicons. The presence of various PCR inhibitors, such as polyphenols, tannins and polysaccharides, may reduce the sensitivity of the qPCR assay. However, this was not the case in this study. We constructed standard curves by diluting DNA extracted with a commercial kit, and we did not detect a significant difference between the waxy and non-waxy samples. The numerous replicates resulted in similar standard curves, demonstrating that the assay is reproducible and highly robust.

The present *waxy* qPCR assay could also be used to determine the proportions of waxy and non-waxy materials in mixed products. After using reference samples to generate standard curves, the absolute quantitative values of waxy and non-waxy DNA could be calculated, and the difference in C_T_ values could be calculated to estimate their proportions in a mixture, as shown in the results. The proportion of *waxy* DNA in a mixed sample could be determined by calculating the cycle differences in the qPCR assays using waxy- and non-waxy-specific primers without further calculation. This calculation should be based on the careful measurement of individual samples, but the ΔC_T_, which can be easily acquired, may be acceptable for most practical applications. This quantitative approach may allow for the detection of very low levels of *waxy* DNA in a sample without first determining the DNA concentration.

*waxy* mutants that affect the function of GBSS have been identified in several small-grain crops such as wheat [18], rice [19,20], barley [21,22], corn [23] and millet [24]; the structures of the non-*waxy* alleles in these cereal species are very similar, but the *waxy* mutant alleles in each species are caused by a variety of mutations such as a point mutations, deletions and the insertion of transposable elements. Because of the structural similarity between target sequences, mutant alleles with deletions or insertions could be analyzed in a manner similar to that used for sorghum. Provided that the target polymorphism was previously validated via sequencing, this assay method could be applied to species for which a large number of sub-species, varieties or hybrids are present in the market.

## Conclusions

This study demonstrates that the presence and quantity of waxy-grain sorghum or waxy-grain sorghum powder in mixed cereal products can be evaluated using quantitative real-time PCR with *waxy* allele-specific primers. Three different primer pairs were used, and each pair specifically amplified a product from only plants containing the target allele. This specificity enabled us to identify *waxy* and non-*waxy* genotypes and estimate their proportions in mixed samples containing both waxy and non-waxy sorghum grains or powder. Our method could be applied to other cereal crops that have similar *waxy* mutant alleles using primers that are similar to those used in this study. This method also provides a diagnostic tool to measure the waxy sorghum content declared on the labels of commercial cereal products.

## Methods

### Plant materials and DNA extraction

Seeds of the Korean sorghum landraces were obtained from the Rural Development Administration (RDA) Genebank Information Center (Suwon, Rep. of Korea), and the Korean sorghum varieties were acquired from the National Institute of Crop Science (Miryang, Rep. of Korea). Genomic DNA from all sorghum accessions was extracted from two- to three-week-old leaves, and for the commercial sorghum products, DNA was extracted from the sorghum grains or powder. All DNA extraction procedures were conducted using a DNeasy Plant Mini kit (QIAGEN, Valencia, California, USA) according to the manufacturer’s instructions.

### PCR analysis of the three waxy alleles

Three *waxy* allele markers were designed based on DNA markers used in previous articles [5,9]; the PCR primers used to detect *wx*^*a*^, *wx*^*b*^ and *wx*^*c*^ were *wx*^*a*^-F, 5′-CGTGGCGAGATCAAACTCTA-3′*; Wx*-F, 5′-GGCCTGGATTCAATGTTCTT-3′; *Wx*-R (which can also be used as *wx*^*a*^-R), 5′-GCAGCTGGTTGTCCTTGTAG-3′; *wx*^*b*^-F, 5′-CGACCGTGTGTTCATTGACCAC-3′; *wx*^*b*^-R, 5′-TTGTTCAGTGCCTTTGCCTCG-3′; *wx*^*c*^-F, GCTGGTTCTGAGTGCAACAA; *wx*^*c*^WT-R, 5′-ACTTCTTCTTGCCAGTGACC-3′ (for the wild type); and *wx*^*c*^-R, 5′-ACTTCTTCTTGCCAGTGACG-3′ (for *wx*^*c*^). The PCR reactions were performed in a final volume of 10 μl and contained 1 μl (5 ng) of DNA template, primers (0.5 μM each), 25 nM dNTPs, 10 X buffer with MgCl_2_, and 2.5 U of *Taq* DNA polymerase (iNtRON Biotechnology, Seongnam-si, Rep. of Korea). The PCR conditions were an initial denaturation at 95°C for 5 min; 40 cycles of denaturation at 95°C for 20 s, annealing at 60°C for 30 s, and extension at 72°C for 50 s; and a final extension at 72°C for 7 min. All PCR amplifications were performed in a GeneAmp PCR System 9700 (Applied Biosystems, Foster City, CA). The PCR products were analyzed by 3% (wt/vol) agarose gel in 1 X TBE electrophoresis buffer containing 0.1 ppm RedSafe^™^ Nucleic Acid Staining Solution (Chembio Ltd., Hertfordshire, England). SiZer^™^-100 DNA Marker Solution (iNtRON Biotechnology, Seongnam-si, Rep. of Korea) was used as the DNA size marker.

### Iodine test (I2–KI staining)

Seeds from self-fertilized individuals from the indicated sorghum accessions were cracked and stained with iodine solution (0.2% I_2_–2% KI). Endosperm that was reddish-brown or dark blue in color was scored as waxy or non-waxy, respectively [25].

### Apparent amylose content

Sorghum grains (100 mg) were milled in 1 ml of ethanol, shaken for 15 min and incubated with 9 ml of 1 N NaOH for 20 min at 95°C. Five ml of the mix was transferred to a 100-ml flask and incubated with 1 ml of 1 N CH_3_COOH and 2 ml of iodine solution (2% KI solution + 0.2% iodine) for 30 min at 30°C. The apparent amylose content of the solution was measured at 620 nm using a UV-2450 (Shimadzu Co., Kyoto, Japan); this technique is based on the colorimetric measurement of the iodine–starch complex (absorbance at 620 nm) as described previously [26]. The ‘M 162’, ‘Dongjin’ and ‘Koami’ rice cultivars were used as controls. The starch from the sorghum grains was analyzed in triplicate.

### qPCR primer design

Three primers were designed based on the sequences flanking the insertion in the third exon of the sorghum *GBSS I* gene: non-*waxy*-specific (*Wx*only), *waxy*-specific (*wx*^*a*^) and non-specific (*Wx*comn). The *Wx*only primers were designed to only amplify a PCR product from the DNA of non-*waxy* plants. Because the *wx*^*a*^ primers were designed based on the insertion sequence, they can be used for only *waxy* plants. The 162 bp at the 3′ end of the insertion, which were used to design the *waxy*-specific primers, were determined by sequencing the PCR product amplified by the *wx*^*a*^ primers [5]. The *Wx*comn primers, which amplified DNA from both waxy and non-waxy plants, were designed based on the *GBSS I* sequence located 83 bp downstream of the insertion. Primer design was performed using Primer3web version 4.0.0 (http://primer3.ut.ee/) with the min and max primer Tm changed to 58 and 60°C, respectively. Information on all eleven primer pairs is listed in Figure 4. A BLAST search (Basic Local Alignment Search Tool, http://blast.ncbi.nlm.nih.gov/Blast.cgi) was conducted to estimate the specificity of each primer. Real-time PCR amplification was performed with an Applied Biosystems 7300 Real Time PCR System (Foster City, California, USA).

### qPCR conditions and test of amplification efficiency

qPCR was performed using an Applied Biosystems 7300 Real Time PCR System in an optical 96-well plate covered with optical sealing tape (Applied Biosystems, Foster City, California, USA). Polymerase chain reactions were carried out in a final volume of 20 μl, which contained 10 μl of SYBR Select Master Mix (Applied Biosystems, Foster City, California, USA), 5 ng of template DNA and primers (0.4 μM each). In addition, to detect genomic DNA contamination, control PCRs with no template (NTC) were also prepared for each primer pair. The reaction mixtures were incubated for a 10 min initial denaturation at 95°C, followed by 40 amplification cycles of 15 s at 95°C and 60 s at 60°C. All reactions were performed in duplicate for each template DNA sample. Following qPCR amplification, to confirm the presence of a single product and the absence of primer dimers, melting curve analysis from 55 to 95°C was conducted with a temperature gradient of 0.5°C every 10 s. The presence of a single qPCR product was further verified by 3% agarose gel electrophoresis.

### Standard curves

Standard curves were generated by plotting the cycle threshold (C_T_) values of the qPCR reactions from serially diluted DNA. Two different genomic DNA samples (*waxy* and non-*waxy*) were two-fold serially diluted, and the dilutions were used to construct standard curves for the *wx*^*a*^, *Wx*only and *Wx*comn primer pairs. The C_T_ value for each dilution was plotted against the log of the template DNA concentration. The qPCR efficiency and regression coefficient (*R*^*2*^) for each primer pair were acquired via the standard curves generated using this method.

### Quantification of mixed cereal grain samples

Mixed grain samples were prepared using various proportions of powders from *waxy* and non-*waxy* sorghum grains (0, 0.5, 1, 2, 5, 10, 20, 50, or 100% *waxy* grains, with the remainder from non-*waxy* grains). The samples were prepared by weighing the grains in the given ratio, evenly grinding with a mortar and pestle and extracting DNA. Mixed DNA samples were also prepared by directly combining DNA from *waxy* and non-*waxy* sorghum in the same ratios as mixed grain powder samples. Commercial cereal products were purchased from the grocery store and used for DNA extraction to confirm the applicability of the qPCR method developed in this study.

The proportion of waxy sorghum in mixtures of waxy and non-waxy sorghum was calculated as follows: the slopes and intercepts of the standard curves described above were calculated for both the waxy and non-waxy samples. qPCR amplification of DNA from the cereal mixtures was carried out in triplicate, and the mean C_T_ values were interpolated in the corresponding standard curve to obtain the DNA content of the sample. Then, the ratio of the samples in the mixture was calculated as

$$ \%\ \mathrm{waxy} = 100\times \left[\mathrm{Waxy}\ \mathrm{D}\mathrm{N}\mathrm{A}\ \mathrm{content}/\left(\mathrm{Waxy}\ \mathrm{D}\mathrm{N}\mathrm{A} + \mathrm{n}\mathrm{o}\mathrm{n}-\mathrm{waxy}\ \mathrm{D}\mathrm{N}\mathrm{A}\right)\right] $$

## Additional files

Additional file 1:
**List of Korean sorghum varieties and landraces and foreign sorghum varieties used in this study.**
Additional file 2:
**Standard curves derived from qPCR using primer pairs specific for** ***waxy*** **and non-** ***waxy*** **genomic DNA or that were nonspecific (amplified both varieties).** For each genomic DNA sample, 100.0 ng of DNA was serially diluted two-fold and amplified using a specific primer pair, and the results were used to draw a standard curve. Three symbols–one for each replicate–are displayed at each DNA concentration. The correlation coefficients were calculated to verify the reliability and efficiency of the qPCR reactions. (A) The results of using the *Wx*comn primer pair on non-*waxy* sorghum DNA, (B) the results of using the *Wx*comn primer pair on *waxy* sorghum DNA, (C) the results of using the *wx* ^*a*^-specific primer pair on *waxy* sorghum DNA, and (D) the results of using the *Wx*only primer pair on non-*waxy* sorghum DNA.

## Abbreviations

GBSS I: Granule-Bound Starch Synthase I
qPCR: Quantitative real-time PCR
NTC: Non-template control
C_T_: Cycle threshold
