# Supplementary figures and images for: Development of a waxy gene real-time PCR assay for the quantification of sorghum waxy grain in mixed cereal products
Source: BMC Biotechnol. 2015 Mar 19;15:20. doi: 10.1186/s12896-015-0134-z (PMC4372279; doi:10.1186/s12896-015-0134-z)

## Slide 1
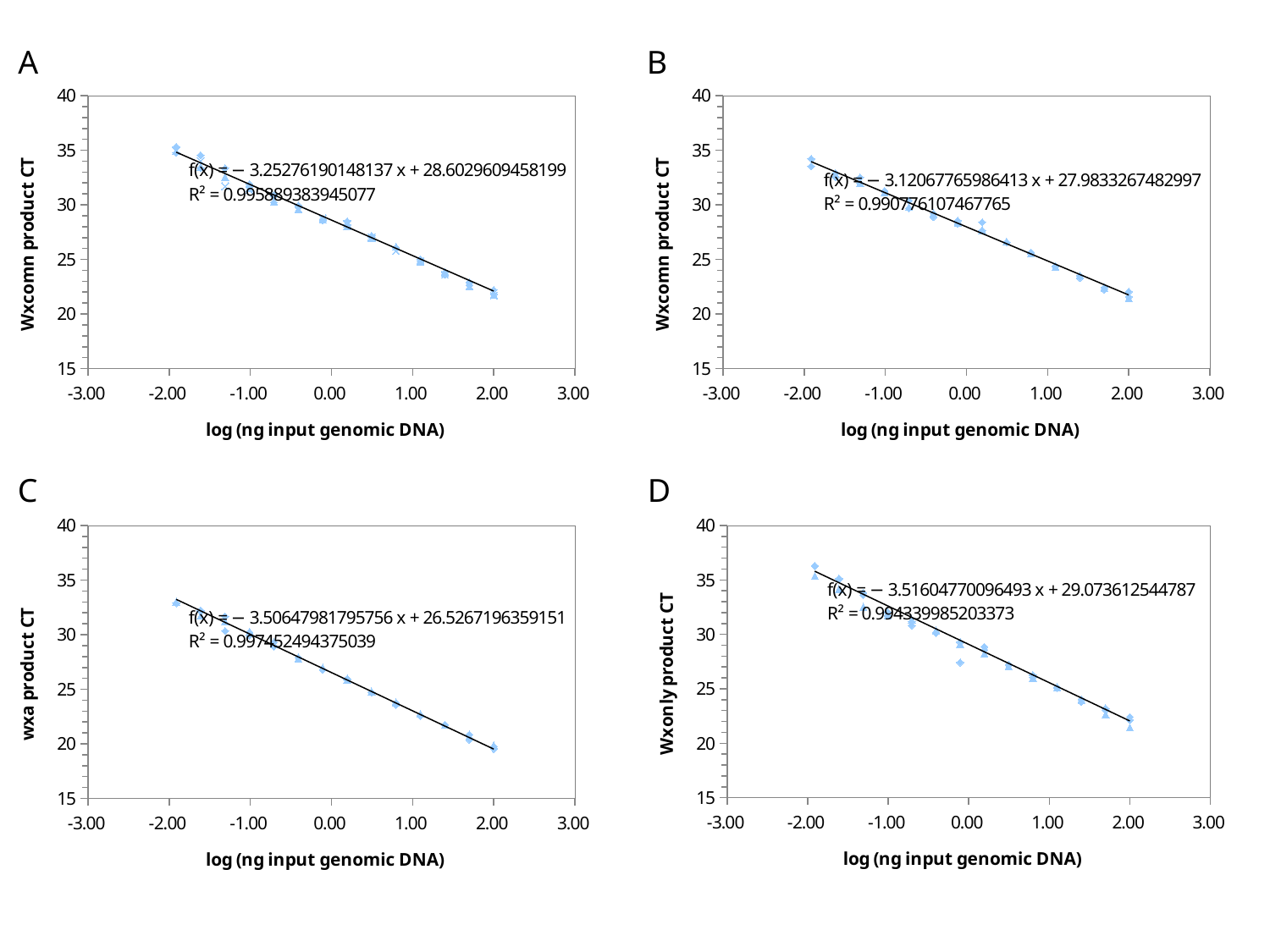

A
B
### Chart
| Category | | | | |
|---|---|---|---|---|
### Chart
| Category | | | |
|---|---|---|---|C
D
### Chart
| Category | | | |
|---|---|---|---|
### Chart
| Category | | | |
|---|---|---|---|

Supplement: Additional file 2: — Standard curves derived from qPCR using primer pairs specific for waxy and non- waxy genomic DNA or that were nonspecific (amplified both varieties). For each genomic DNA sample, 100.0 ng of DNA was serially diluted two-fold and amplified using a specific primer pair, and the results were used to draw a standard curve. Three symbols–one for each replicate–are displayed at each DNA concentration. The correlation coefficients were calculated to verify the reliability and efficiency of the qPCR reactions. (A) The results of using the Wxcomn primer pair on non-waxy sorghum DNA, (B) the results of using the Wxcomn primer pair on waxy sorghum DNA, (C) the results of using the wx a-specific primer pair on waxy sorghum DNA, and (D) the results of using the Wxonly primer pair on non-waxy sorghum DNA. [file 12896_2015_134_MOESM2_ESM.pptx]
